# Exploring Possible Links: Thigh Muscle Mass, Apolipoproteins, and Glucose Metabolism in Peripheral Artery Disease—Insights from a Pilot Sub-Study following Endovascular Treatment

**DOI:** 10.3390/metabo14040192

**Published:** 2024-03-29

**Authors:** Takeshi Ikeda, Hidenori Komiyama, Tomoyo Miyakuni, Masamichi Takano, Kuniya Asai

**Affiliations:** 1 Cardiovascular Medicine, Nippon Medical School, Tokyo 113-8603, Japan; ikedata@nms.ac.jp (T.I.); kasai@nms.ac.jp (K.A.); 2 Cardiovascular Medicine, Saitama Medical Center, Saitama Medical University, Saitama 350-8550, Japan; 3 Cardiovascular Medicine, Nippon Medical School Chiba Hokusoh Hospital, Chiba 270-1613, Japan; tomoyo-m@nms.ac.jp (T.M.);

**Keywords:** apolipoproteins, endovascular treatment, glucose metabolism, peripheral artery disease, risk factors, skeletal muscle

## Abstract

Peripheral artery disease (PAD) compromises walking and physical activity, which results in further loss of skeletal muscle. The cross-sectional area of the thigh muscle has been shown to be correlated with systemic skeletal muscle volume. In our previous pilot study, we observed an increase in thigh muscle mass following endovascular treatment (EVT) in patients with proximal vascular lesions affecting the aortoiliac and femoropopliteal arteries. Considering the potential interactions between skeletal muscle, lipid profile, and glucose metabolism, we aimed to investigate the relationship between thigh muscle mass and apolipoproteins as well as glucose metabolism in PAD patients undergoing EVT. This study is a prespecified sub-study conducted as part of a pilot study. We prospectively enrolled 22 symptomatic patients with peripheral artery disease (PAD) and above-the-knee lesions, specifically involving the blood vessels supplying the thigh muscle. The mid-thigh muscle area was measured with computed tomography before and 6 months after undergoing EVT. Concurrently, we measured levels of apolipoproteins A1 (Apo A1) and B (Apo B), fasting blood glucose, 2 h post-load blood glucose (using a 75 g oral glucose tolerance test), and glycated hemoglobin A1c (HbA1c). Changes in thigh muscle area (delta muscle area: 2.5 ± 8.1 cm^2^) did not show significant correlations with changes in Apo A1, Apo B, fasting glucose, 2 h post-oral glucose tolerance test blood glucose, HbA1c, or Rutherford classification. However, among patients who experienced an increase in thigh muscle area following EVT (delta muscle area: 8.41 ± 5.93 cm^2^), there was a significant increase in Apo A1 (pre: 121.8 ± 15.1 mg/dL, 6 months: 136.5 ± 19.5 mg/dL, *p* < 0.001), while Apo B remained unchanged (pre: 76.4 ± 19.2 mg/dL, 6 months: 80.5 ± 4.9 mg/dL). Additionally, post-oral glucose tolerance test 2 h blood glucose levels showed a decrease (pre: 189.7 ± 67.5 mg/dL, 6 months: 170.6 ± 69.7 mg/dL, *p* = 0.075). Patients who exhibited an increase in thigh muscle area demonstrated more favorable metabolic changes compared to those with a decrease in thigh muscle area (delta muscle area: −4.67 ± 2.41 cm^2^). This pilot sub-study provides insights into the effects of EVT on thigh muscle, apolipoproteins, and glucose metabolism in patients with PAD and above-the-knee lesions. Further studies are warranted to validate these findings and establish their clinical significance. The trial was registered on the University Hospital Medical Information Network Clinical Trials Registry (UMIN000047534).

## 1. Introduction

Skeletal muscles impact lipid and glucose metabolism, and vice versa [1,2]. This interplay significantly affects the development and progression of atherosclerosis. Changes in skeletal muscle mass and function, whether through increases or decreases, are closely linked to patients’ susceptibility to cardiovascular events and overall mortality [3].

Engaging in physical activity utilizing skeletal muscles [4] directly impacts lipid metabolism [1,2]. In individuals with established coronary artery disease on statin therapy, blood levels of Apo A1 and Apo B predict myocardial infarction and overall mortality, making them more suitable markers for assessing cardiovascular risk [5]. Among the lipid and glucose parameters associated with atherosclerosis, glycated hemoglobin A1c (HbA1c)/Apo A1 [6] and Apo A1/Apo B [7] have emerged as markers for evaluating atherosclerotic status.

Impaired glucose tolerance is a significant risk factor for all-cause mortality and cardiovascular events in the context of atherosclerosis [8]. Evaluation of glucose tolerance is commonly performed through the oral glucose tolerance test (OGTT), which measures blood glucose levels two hours after a 75 g glucose load. Impaired OGTT has a stronger association with atherosclerosis compared to fasting blood glucose (FBG) or HbA1c levels [9]. Skeletal muscles, known for their role in glucose storage and utilization [10], may have a close relationship with glucose metabolism.

Apo A1 has been reported to promote glucose consumption and enhance glucose uptake in skeletal muscles, thus improving glucose tolerance [11,12]. Conversely, elevated blood glucose levels downregulate apolipoprotein M expression, which in turn affects Apo A1 levels [13,14]. This creates a bidirectional relationship between Apo A1 and diabetes, perpetuating a vicious cycle [15]. 

In a preliminary report, we demonstrated changes in ischemic thigh muscle volume in patients with above-the-knee PAD before and after endovascular treatment (EVT) for occlusive iliofemoral arteries supplying the thigh muscle [16]. PAD itself hampers physical activity, leading to a sedentary lifestyle that worsens blood sugar elevation and which induces skeletal muscle loss through inflammatory pathway activation [17,18]. Management of PAD involves improving intermittent claudication (IC) symptoms and promoting physical activity post-EVT [19]. The severity of IC is commonly assessed using the Rutherford classification [20]. However, the relationships between metabolic parameters and skeletal muscle mass in PAD is still not well understood.

In the broader context of PAD management, the interplay between metabolic health and physical capacity remains an area ripe for exploration. While this study does not directly measure physical activity, acknowledging its general role in cardiovascular health underscores the importance of a multifaceted approach to PAD treatment. This includes understanding the potential impacts of lifestyle factors alongside medical interventions, such as EVT. Our research thus situates itself within this complex landscape, aiming to illuminate how metabolic changes post-EVT might reflect broader health outcomes in PAD patients.

In addressing the intricate dynamics between metabolic factors and structural changes in patients with above-the-knee PAD undergoing EVT, our sub-study, conducted within the context of a pilot investigation, posits a focused hypothesis: significant associations are anticipated between changes in Apo A1 and Apo B levels, glucose metabolism, and alterations in thigh muscle area post-EVT, reflecting the clinical progression of PAD. This hypothesis is grounded in the documented but underexplored interplay between lipid and glucose metabolism and PAD’s clinical outcomes, aiming to illuminate potential therapeutic targets and intervention strategies.

The rationale for undertaking this study emerges from a critical gap in understanding how EVT affects not only vascular conditions but also metabolic and structural parameters in PAD patients. Despite advancements in PAD management, the comprehensive impact of these interventions on broader health outcomes remains insufficiently explored. By examining these associations, our research endeavors to contribute valuable insights that could inform more nuanced and effective PAD management strategies, ultimately enhancing patient care and outcomes.

This exploration is pivotal, offering potential pathways for integrating metabolic and structural considerations into the broader treatment landscape for PAD, thus addressing both the specific scientific inquiry and the broader clinical implications.

## 2. Materials and Methods

### 2.1. Study Design

This pilot sub-study was conducted as a predefined component of the original pilot study [16]. The study design, including the cohort, year of study, sample size, and inclusion/exclusion criteria, was identical to the main study. It was a single-center, prospective, observational pilot study conducted at Nippon Medical School Chiba Hokusoh Hospital. Patients with consecutive lower extremity above-the-knee iliofemoral PAD and symptomatic IC, which was classified according to the Rutherford criteria, were prospectively enrolled. The recruitment and follow-up period spanned from April 2016 to March 2020. All patients underwent EVT for iliofemoral lesions. To minimize the potential confounding effects of medication, including anti-diabetic drugs and statins, prescribed drug treatment was initiated at least one month prior to study enrollment and continued throughout the study duration. Patients receiving maintenance hemodialysis and those with critical limb ischemia were excluded from this study due to its association with excessive skeletal muscle loss [21,22]. 

### 2.2. Ethics

Ethical approval for this study was obtained from the Institutional Review Board of Nippon Medical School Chiba Hokusoh Hospital (IRB number: 504) on 25 April 2016. The trial was registered in the University Hospital Medical Information Network Clinical Trials Registry (UMIN000047534). The study adhered to the ethical standards in the Declaration of Helsinki 1975 and obtained signed informed consent from all participating patients prior to inclusion.

### 2.3. Endovascular Treatment

Patients with medication or contrast agent intolerance were excluded. During the procedure, weight-adjusted intravenous heparin was administered to achieve a target-activated clotting time of over 300 s. Balloon dilation and stent placement were performed based on the operator’s assessment of flow limitation and residual stenosis of more than 30% in the target vessels. EPIC stents (Boston Scientific, Marlborough, MA, USA) and SMART stents (Cordis Co., Miami Lakes, FL, USA) were utilized. Vascular patency following EVT was assessed using the ankle–brachial index (ABI) value, which was monitored during the study.

### 2.4. Measurements of the Thigh Muscle Area, Apo A1, B, OGTT, and Glycated HbA1c

**Figure 1:**
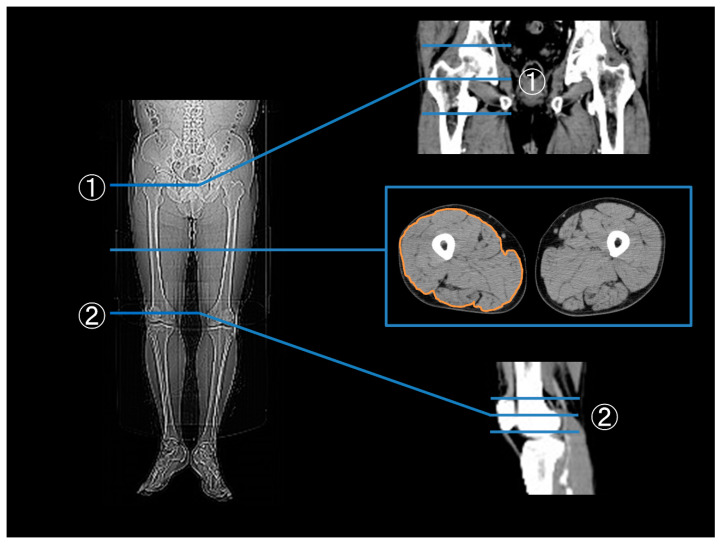
Measurement of the mid-thigh muscle area. The thigh muscle area was measured at the mid-point cross-sections of 1 and 2; (1) Upper edge: mid-point of the upper margin of the greater trochanter and the lower margin of the femoral condyles, (2) Lower edge: mid-point of the upper margin of the patella and the lower margin of the patella.

In a previous study, skeletal muscle measurements were assessed using a single-slice CT scan to determine muscle volume [23]. A 64-slice CT scan of the legs was conducted before and six months after EVT utilizing the Toshiba Aquilion 64 system (Toshiba Medical Systems, Otawara, Japan) to evaluate the effects of the procedure. The cross-sectional area of the mid-thigh, was measured between the pubic symphysis and the inferior condyle of the femur [24]. Thigh muscle area was analyzed using the SYNAPSE commercial workstation (Fujifilm, Tokyo, Japan) (SYNAPSE; Fujifilm, Tokyo, Japan) (Figure 1). Based on the changes in skeletal muscle cross-sectional area before and after EVT, patients were categorized into skeletal muscle gain or loss groups. 

The concomitant serum Apo A1 and Apo B were analyzed using immune nephelometry as secondary measures. Both assays had a percentage coefficient of variation (CV) ≤ 5%. Measurements were taken on admission and at six months after EVT using the Sekisui Medical Ltd. assay (Tokyo, Japan). HbA1c/Apo A1 and Apo B/Apo A1 numeric values were calculated based on the measured concentrations. Glycated HbA1c levels were measured for glucose control (normal limit < 6.5%, 47 mmol/mol). An oral glucose tolerance test (OGTT) was performed to assess glucose tolerance, with blood samples taken before and at 30 min, 60 min, and 120 min after ingesting 75 g of glucose solubilized in 250–300 mL of water. The OGTT was conducted at admission and six months after EVT. Exploratory analyses of the atherosclerotic metrics Apo B/Apo A1 and HbA1c/Apo A1 were performed using the collected data.

### 2.5. ABI Measurement

The ABI was determined using a Colin wave-form analyzer (BP-203RPE III; Omron Colin, Tokyo, Japan), adhering to the guidelines proposed by the American Heart Association [25]. ABI was measured on admission and then again at six months after discharge. 

### 2.6. Statistical Analyses

Sample size calculations were not conducted for this exploratory pilot study. However, a sample size of sixteen was determined to achieve a statistical power of 80% and a significance level of 5% (two-sided) for detecting an effect size of 0.8 in paired differences. Descriptive statistics were used to present categorical and continuous variables. The Shapiro–Wilk test was used to assess the normal distribution of variables, and for dichotomous variables, the chi-square test was employed, or Fisher’s exact test was used when there were fewer than 5 instances. Spearman correlation analysis investigated possible correlations between the changes in thigh muscle area after EVT, Apo A1 and B concentrations, fasting glucose (FBG), post 2 h blood glucose (OGTT-2hBG), HbA1c, and Rutherford classification. Single-tailed paired t-tests compared concentrations of apolipoproteins and glucose, as well as exploratory analyses of atherosclerotic parameter metrics. Statistical power analysis was conducted using the PWR package [26], and all statistical analyses were performed using R software (ver 3.6.2). A significance level of 0.05 was used.

## 3. Results

**Figure 2:**
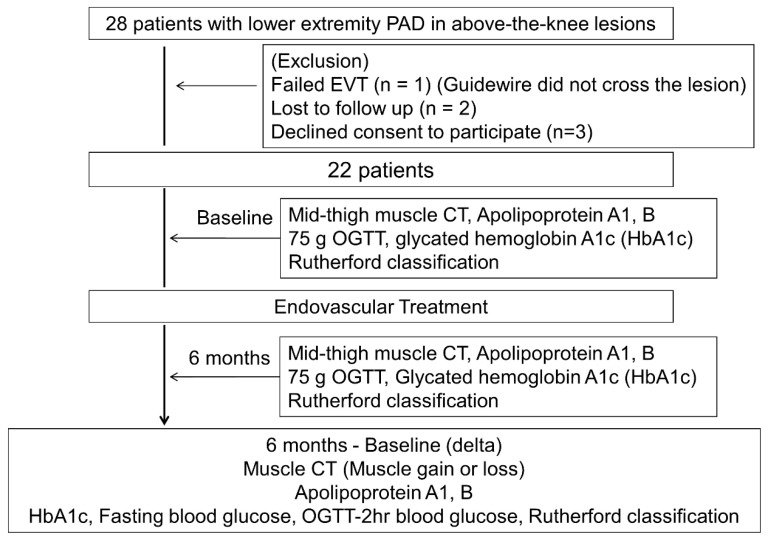
Study design flowchart. A total of 28 patients were enrolled and 22 patients completed the study. Patients had received thigh muscle CT, taken apolipoprotein (A1 and B), fasting blood for measurement of glycated hemoglobin (HbA1c), and 75 g oral glucose tolerance test (75 g OGTT). Delta values (6 months-baseline) of each parameters were further evaluated.

Figure 2 illustrates the flowchart depicting the study design. 

Initially, a total of 28 patients were enrolled in the study in a prospective manner. Out of these, EVT was unsuccessful in one patient, two patients were lost to follow-up, and three patients declined to provide consent. Consequently, the study was completed with 22 patients who successfully underwent EVT and were included in the final analysis.

Table 1 presents the summarized clinical characteristics of the study’s participants. The average age of the patients was 72.4 ± 7.4 years. Most patients were male (91%), and certain characteristics, such as hypertension (82%) and dyslipidemia (86%), were prevalent. The initial clinical symptoms were categorized based on the Rutherford PAD classification, consisting of category 1 (11 patients, 50.00%), category 2 (10 patients, 45.45%), and category 3 (1 patient, 4.55%). Prior to undergoing EVT, dual antiplatelet therapy was administered to all participants. Table 2 details the lesion locations and characteristics. The analysis revealed no significant differences in the gain or loss of skeletal muscle across the lesion sites. Post-EVT, clinical symptoms were noted to improve, a trend that persisted over the course of the study. At the six-month post-EVT mark, 82% (18 patients) were reclassified into category 0, with the remaining 18% (4 patients) falling into category 1. The day following EVT saw the successful discharge of all patients, with no reported complications. Throughout the duration of the study, vascular patency was consistently monitored and confirmed via ABI values post-EVT (30 ischemic lower limbs: pre-EVT 0.79 ± 0.14, post-EVT at 6 months 1.06 ± 0.16; 14 non-ischemic lower limbs: pre-EVT 1.01 ± 0.10, post-EVT at 6 months 1.05 ± 0.14).

### 3.1. Thigh Muscle Area before and after EVT

The thigh muscle area of each patient was reported previously [16] and altered after EVT (delta muscle area: 2.5 ± 8.1 cm^2^, n = 22). Of these, 12 patients gained skeletal muscle (positive change in thigh muscle area: 8.4 ± 5.9 cm^2^), and 10 patients lost skeletal muscle (negative change in thigh muscle area: −4.7 ± 2.4 cm^2^). These changes were based on comparisons of skeletal muscle area measurements taken before EVT and six months afterwards (Table 3). 

### 3.2. Correlation Analysis among the Changes in Apo A1, B, Glucose Metabolism, Intermittent Claudication Symptom, and Skeletal Muscle Mass

**Figure 3:**
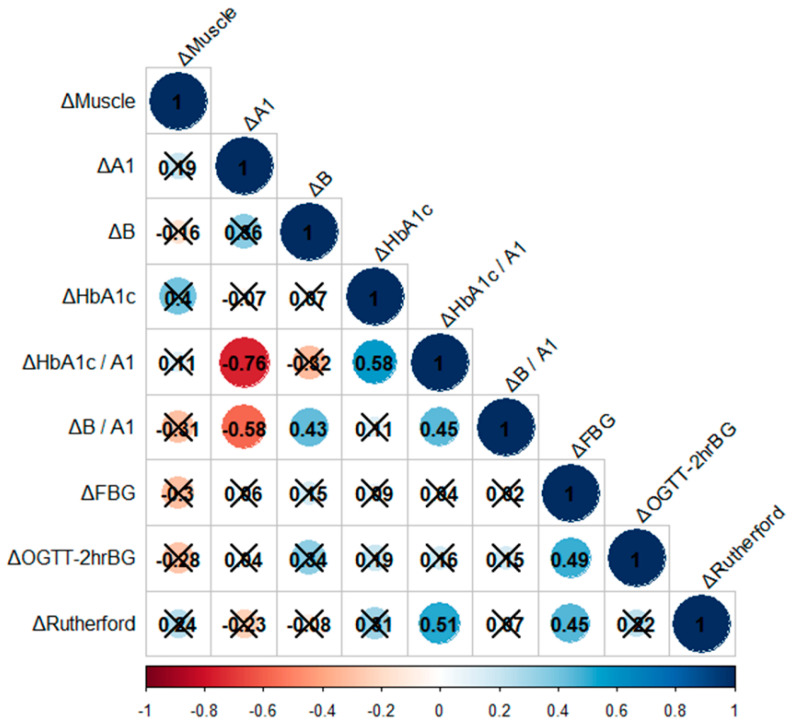
Correlation matrix between delta values of Apo A1, B, fasting glucose, post 2 h -blood glucose, HbA1c, Rutherford classification, and delta thigh muscle area after EVT. A correlation matrix between delta values is drawn based on Spearman correlation analysis. Correlation coefficients are colored according to the value combined with significant values, and *p*-values ≥ 0.05 were considered insignificant and have been crossed out.

Correlation analyses were conducted to explore the relationships between the delta thigh muscle area after EVT, changes in the delta values of Apo A1 and B, fasting glucose, post 2 h -blood glucose, HbA1c, and Rutherford classification. However, no significant correlations were found between delta muscle area and the delta values of the other parameters (Figure 3). 

Similar correlation analyses were performed separately for patients with muscle gain (Supplementary Figure S1) and those with muscle loss (Supplementary Figure S2). 

Within the assessed variables, a negative correlation was observed between change in Apo A1 and change in HbA1c/Apo A1 ratio (R = −0.76, *p* < 0.01) as well as change in parameter Apo B (R = −0.58, *p* = 0.005). Conversely, an increase in Apo B showed a positive association with the Apo B/Apo A1 ratio (R = 0.43, *p* = 0.005). Similarly, an increase in HbA1c was positively associated with the HbA1c/Apo A1 ratio (R = 0.58, *p* < 0.01). The change in the HbA1c/Apo A1 ratio also showed a positive relationship with the change in the Apo B/Apo A1 ratio (R = 0.45, *p* = 0.038) and changes in the Rutherford classification for peripheral arterial disease (R = 0.51, *p* = 0.015). Additionally, a positive correlation was found between changes in fasting blood glucose (FBG) and changes in 2 h blood glucose during an oral glucose tolerance test (OGTT-2h BG) (R = 0.49, *p* = 0.019), as well as changes in the Rutherford classification (R = 0.45, *p* = 0.034) (Figure 3).

### 3.3. Glucose Tolerance and Control before and after EVT

**Figure 4:**
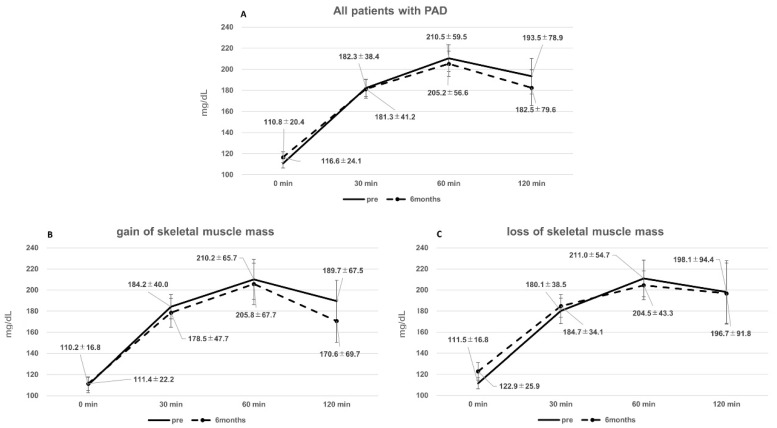
Results of the 75-g OGTT. (**A**) In all the patients, the blood glucose level did not change during fasting, 30 min after OGTT, 60 min after OGTT, and 2 h after OGTT. (**B**) In patients with gain in skeletal muscle, the blood glucose level did not change during fasting, 30 min after OGTT, and 60 min after OGTT, but decreased 2 h after OGTT. (**C**) In patients with loss of skeletal muscle, the fasting glucose level increased, but there was no alteration in 30 min after OGTT, 60 min after OGTT, and 2 h after OGTT.

Overall, there were no significant changes observed in blood glucose levels during fasting (pre: 110.8 ± 20.4 mg/dL, 6 months: 116.6 ± 24.1 mg/dL) and 2 h after OGTT (pre: 193.5 ± 78.9 mg/dL; 6 months: 182.5 ± 79.6 mg/dL) (Figure 4). 

HbA1c levels also remained stable (pre: 6.1 ± 0.4%, 6 months: 6.2 ± 0.4%), and there were no significant differences between patients exhibiting an increase in skeletal muscle (pre: 5.8 ± 0.4%, 6 months: 6.0 ± 0.4%) and those exhibiting a decrease in skeletal muscle (pre: 6.5 ± 0.8%, 6 months: 6.5 ± 1.1%) (Table 2).

Among patients exhibiting an increase in skeletal muscle, fasting glucose levels did not change significantly (pre: 110.2 ± 25.0 mg/dL, 6 months: 111.4 ± 22.2 mg/dL). However, there was a tendency for a decrease in blood glucose levels 2 h after OGTT (pre: 189.7 ± 67.5 mg/dL, 6 months: 170.6 ± 69.7 mg/dL, *p* = 0.0754) (Figure 4B). In contrast, patients with skeletal muscle loss showed a tendency for an increase in fasting glucose levels (pre: 111.5 ± 16.8 mg/dL, 6 months: 122.9 ± 25.9 mg/dL, *p* = 0.0939), while blood glucose levels 2 h after OGTT were unchanged (pre: 198.1 ± 94.4 mg/dL, 6 months: 196.7 ± 91.8 mg/dL) (Figure 4C).

### 3.4. Changes in Apo A1 and B before and after EVT

Overall, Apo A1 levels significantly increased after EVT (pre: 119.0 ± 17.4 mg/dL, 6 months: 129.6 ± 19.6 mg/dL, *p* = 0.0027) (Table 3). Specifically, patients exhibiting an increase in skeletal muscle showed a significant increase in Apo A1 levels (pre: 121.8 ± 15.1 mg/dL, 6 months: 136.5 ± 19.5 mg/dL, *p* < 0.001) (Table 4). However, there were no significant changes observed in Apo B levels for both patients exhibiting an increase in skeletal muscle (pre: 76.4 ± 19.2 mg/dL, 6 months: 80.5 ± 4.9 mg/dL) and those exhibiting a decrease in skeletal muscle (pre: 78.6 ± 19.8 mg/dL, 6 months: 82.3 ± 19.3 mg/dL) (Table 4).

### 3.5. Atherosclerotic Metrics of Apo B/Apo A1 and HbA1c/Apo A1

In general, there were no significant changes in Apo B/Apo A1 (pre: 0.66 ± 0.18, 6 months: 0.64 ± 0.16) (Table 3). However, there was a tendency for a decrease in patients exhibiting an increase in skeletal muscle (pre: 0.64 ± 0.18, 6 months: 0.60 ± 0.17, *p* = 0.0693), while no significant changes were observed in patients exhibiting a decrease in skeletal muscle (pre: 0.69 ± 0.19, 6 months: 0.68 ± 0.16) (Table 4). On the other hand, HbA1c/Apo A1 significantly decreased overall (pre: 0.053 ± 0.011, 6 months: 0.049 ± 0.011, *p* = 0.0489) (Table 3). The decrease was significant among patients with skeletal muscle gain (pre: 0.049 ± 0.007, 6 months: 0.045 ± 0.008, *p* = 0.0070), but no significant changes were observed in patients with skeletal muscle loss (pre: 0.058 ± 0.013, 6 months: 0.054 ± 0.012) (Table 4).

## 4. Discussion

The main findings of this study are as follows:

1. There was no significant correlation between changes in muscle area and levels of Apo A1, Apo B, glucose metabolism, and Rutherford classification.
2. Apo A1 significantly increased in patients with skeletal muscle gain after EVT, while Apo B did not change in either group.
3. Patients with muscle gain showed improved glucose tolerance, whereas patients with muscle loss had increased fasting glucose levels.

### 4.1. Changes in Apo A1 and B, Glucose Metabolism, Intermittent Claudication Symptom, and Skeletal Muscle Mass

Although no significant correlation was found between changes in muscle area and the levels of Apo A1, Apo B, glucose metabolism, or Rutherford classification, patients with skeletal muscle gain showed significantly increased levels of Apo A1. This increase in Apo A1 levels can be attributed to the promotion of physical activity following successful EVT, as exercise is known to elevate Apo A1 levels [27]. Our study sheds light on the importance of skeletal muscle gain post-EVT and its association with improved metabolic markers in PAD patients. These observations suggest a promising area for further research, particularly regarding the role of Apo A1 and its potential as a marker for clinical improvement.

The study suggests that even modest increases in physical activity, such as walking, after EVT can positively impact metabolism in an anti-atherosclerotic manner [28]. Apo B did not exhibit significant changes in patients with muscle gain or loss, possibly due to the administration of statin therapy to all enrolled PAD patients.

Current clinical practice guidelines recommend statin therapy initiation for preventing subsequent cardiovascular events in established cardiovascular disorders [29]. Studies have demonstrated the efficacy of statin therapy, with on-treatment levels of Apo A1 and Apo B identified as significant predictors of myocardial infarction and all-cause mortality in patients with established coronary artery disease, suggesting their potential in assessing cardiovascular risk [5]. Apo A1, a major component of high-density lipoprotein (HDL), plays a crucial role in cholesterol clearance from artery walls, contributing to the prevention of atherosclerosis progression [30,31]. In contrast, atherogenic Apo B particles trapped within arterial walls initiate and sustain inflammation, ultimately leading to advanced atherosclerosis [32].

In our previous report, we found that patients with PAD and normal glycemic control (HbA1c < 6.5) prior to EVT exhibited an increase in skeletal muscle mass (16). Therefore, patients with skeletal muscle gain after EVT are more likely to have closer-to-normoglycemic levels compared to those with muscle loss. Hyperglycemia negatively affects muscle signaling and exercise capacity, making patients with PAD and high blood glucose levels more susceptible to skeletal muscle loss (17). 

HbA1c is commonly used clinically to monitor glycemic control in diabetic patients [33]. In our study, participants did not restrict their caloric intake or receive dietary guidance, as indicated by unchanged HbA1c values [34]. Glucose tolerance was assessed using a 75-g oral glucose tolerance test (OGTT). Studies have shown that blood glucose levels 2 h after the OGTT are closely associated with atherosclerosis [9]. Disruption of glucose transporters in muscles leads to insulin resistance and glucose intolerance, while exercise upregulates glucose transporters, improving glucose tolerance [35,36]. Although we did not observe a correlation between changes in skeletal muscle volume and glucose metabolism, patients exhibiting an increase in skeletal muscle tended to demonstrate improved glucose tolerance, while those with muscle loss exhibited deteriorated fasting glucose levels. Additionally, Apo A1 is known to improve glucose tolerance [11,12], and patients with skeletal muscle gain showed increased Apo A1 levels along with improved glucose tolerance, consistent with previous reports. Accordingly, physical activity would increase Apo A1 levels and may improve glucose tolerance [27,28]. While our study has not directly measured physical activity, the observed increase in Apo A1 levels and improved glucose tolerance in patients with skeletal muscle gain post-EVT suggests the potential role of increased physical activity. Given the well-documented benefits of exercise on metabolic health, including the elevation of Apo A1 levels and enhancement of glucose utilization, it is plausible to infer that the patients experiencing muscle gain may have engaged in greater physical activity levels. This inference aligns with the existing literature that underscores the importance of physical activity in managing PAD and its metabolic consequences. Consequently, while direct correlations between physical activity and our observed metabolic improvements cannot be definitively established from our data, the implications of these findings highlight the potential significance of physical activity in the recovery and management of PAD patients post-EVT.

### 4.2. Atherosclerosis Indicators: HbA1c/Apo A1 and Apo B/Apo A1

Apolipoproteins serve as biological markers and can exhibit either atherogenic or anti-atherogenic properties. A recent study demonstrated that the HbA1c/Apo A1 ratio after myocardial infarction is predictive of all-cause mortality and major adverse cardiac events, comprising nonfatal myocardial infarction, cardiac death, and revascularization [6]. Additionally, several studies have established the Apo B/Apo A1 ratio as a parameter for assessing atherosclerotic conditions [5]. Recent research has suggested that Apo B and Apo A1 may not be sufficient for evaluating the risk of recurrent cardiovascular events in patients receiving statin treatment [37]. In our present study, there was no significant correlation between changes in skeletal muscle and the delta values of HbA1c/Apo A1 and Apo B/Apo A1. However, we did observe a significant correlation between the delta values of Apo A1 and HbA1c/Apo A1 as well as Apo B/Apo A1. This suggests that Apo A1 plays a role in modifying the parameters of atherosclerosis (HbA1c/Apo A1 and Apo B/Apo A1). Therefore, changes in Apo A1 levels following EVT may impact atherosclerotic conditions. Furthermore, HbA1c/A1 was significantly decreased in patients who experienced muscle gain after EVT. However, it is important to note that previous studies lacked long-term consecutive follow-up, and further research is needed to explore long-term changes.

## 5. Study Limitations

Our study has several limitations. Firstly, despite achieving sufficient statistical power, our study acknowledges that a larger sample size could further enhance the robustness of our results. Additionally, while our research did not specifically focus on the influence of age and sex on PAD treatment outcomes and metabolic responses, we recognize the importance of these factors. Acknowledging the potential variability in treatment outcomes influenced by age and sex, we suggest future investigations to delve into how these factors may affect the metabolic improvements observed in PAD patients post-EVT. Such insights could pave the way for more personalized therapeutic approaches, tailoring treatments to meet individual patients’ needs more effectively. Secondly, while a six-month observation period is sufficient for observing muscle increase, it is short for assessing long-term EVT benefits. Thirdly, we focused on mild claudication patients, excluding those with below-the-knee lesions, on hemodialysis, or with critical limb ischemia. As PAD patients often present at advanced cardiovascular stages [38], early detection is crucial [39]. Fourthly, we excluded patients suffering neuropathic muscle atrophy, common in certain conditions, like diabetic neuropathy [40] and lumbar disc herniation [41], due to overlapping symptoms with PAD. Fifthly, we did not perform duplex ultrasound, a more accurate test for vascular patency. Sixthly, institutional limitations prevented a specific exercise regimen. Lastly, we did not objectively evaluate physical activity, which currently relies on questionnaires and interviews [42,43]. This limitation may influence the interpretation of our findings, particularly in understanding the full impact of physical activity on the metabolic parameters and muscle changes observed in our PAD patient cohort. Future studies could greatly benefit from the incorporation of objective measures of physical activity, such as those provided by wearable devices [44,45], to gain a more comprehensive understanding of its role in PAD management and recovery. Such advancements would enable a more nuanced analysis of the interplay between physical activity levels and the metabolic and structural changes post-EVT, offering clearer insights into optimal PAD treatment strategies.

## 6. Conclusions

This sub-study, part of a pilot study, revealed unexplored correlations between metabolic parameters and skeletal muscle mass. Earlier reports suggested EVT may preserve thigh muscles [16]. Our study confirmed EVT’s positive effects on apolipoproteins and glucose metabolism in PAD patients, with muscle gain post-EVT patients showing increased Apo A1 levels and potential glucose tolerance improvement. More thorough investigations are needed to further understand the regulation of metabolism and skeletal muscle mass.

## Figures and Tables

**Table 1:** Baseline characteristics of the enrolled patients.

	All	Gain of Skeletal Muscle	Loss of Skeletal Muscle	*p*-Value
Number of patients	22	12	10	
Age (years)	72.4 ± 7.4	69.7 ± 7.5	75.6 ± 6.2	0.06
Male	20 (91)	12 (100)	8 (80)	N.S
Female	2 (9)	0 (0)	2 (20)	
Hypertension	18 (82)	10 (83)	8 (80)	N.S
Dyslipidemia	19 (86)	10 (83)	8 (80)	N.S
Diabetes	10 (45)	6 (50)	4 (40)	N.S
HbA1c (%)	6.1 ± 0.7	5.8 ± 0.4 *	6.5 ± 0.8 *	0.0175 *
BMI	24.4 ± 3.5	25.1 ± 2.6	23.5 ± 4.4	N.S
Past smoker	10 (45)	6 (50)	4 (40)	N.S
Rutherford classification	1.5 ± 0.6	1.6 ± 0.5	1.5 ± 0.7	N.S
Medications				
Dual antiplatelet therapy	22 (100)	12 (100)	10 (100)	N.S
Aspirin	19 (86)	10 (83)	9 (90)	N.S
Clopidogrel	9 (41)	5 (42)	4 (40)	N.S
Cilostazol	6 (27)	2 (8)	4 (40)	N.S
Prasugrel	6 (27)	4 (33)	2 (20)	N.S
Statin	21 (95)	11 (92)	10 (100)	N.S
ACE-I/ARB	22 (100)	12 (100)	10 (100)	N.S
β-blocker	15 (68)	8 (67)	7 (70)	N.S
Anti-diabetic drugs	9 (41)	5 (42)	4 (40)	N.S
Voglibose	2 (9)	1 (8)	1 (10)	N.S
Metformin	3 (14)	2 (17)	1 (10)	N.S
Glimepride	1 (5)	0 (0)	1 (10)	N.S
DPP-4 inhibitor	8 (36)	5 (42)	3 (30)	N.S
SGLT-2 inhibitor	1 (5)	0 (0)	1 (10)	N.S
Thiazolidine	1 (5)	0 (0)	1 (10)	N.S
Glinide	2 (9)	1 (8)	1 (8)	N.S

Data are presented as numbers (%). *p* * < 0.05, Not significant (N. S). ACE-I, angiotensin-converting enzyme inhibitor; ARB, angiotensin II receptor blocker. DPP-4, dipeptidyl peptidase-4; SGLT-2, sodium-glucose cotransporter 2; statin regimen is listed in Supplementary Table S1.

**Table 2:** Location and characteristics of lesions.

Variables	All	Gain of Skeletal Muscle	Loss of Skeletal Muscle	*p*-Value
Location of lesions	N = 30	N = 17	N = 13	
Unilateral lesion Bilateral lesions	14 (64) 8 (36)	7 (58) 5 (42)	7 (70) 3 (30)	N.S
Iliac artery	16 (53)	10 (59)	6 (46)	N.S
Superficial femoral artery	9 (30)	6 (35)	5 (38)	N.S
Common femoral artery	1 (3)	0 (0)	1 (8)	N.S
Popliteal artery	3 (13)	1 (6)	0 (0)	N.S
Superficial + popliteal artery	1 (5)	0 (0)	1 (8)	N.S
TASC II classification				
Type A	17 (58)	10 (59)	7 (54)	N.S
Type B	6 (20)	3 (18)	3 (23)	N.S
Type C	2 (6)	0 (0)	2 (15)	N.S
Type D	5 (16)	4 (23)	1 (8)	N.S
Stent implantation	24 (80)	15 (88)	9 (70)	N.S

Data are presented as numbers (%). N.S, not significant; TASC, Trans-Atlantic Intersociety Consensus.

**Table 3:** Change in muscle area, apolipoprotein A1, apolipoprotein B, glucose metabolism, and Rutherford classification.

All (n = 22)	Before	After 6 Months	*p*-Value	Delta Value
Muscle area (cm^2^)	239.8 ± 34.8	243.1 ± 40.8	0.21	2.5 ± 8.1
Apo A1 (mg/dL)	119.0 ± 17.4 **	129.6 ± 19.6 **	0.0027 **	10.7 ± 14.7
Apo B (mg/dL)	76.3 ± 17.3 *	81.3 ± 17.7 *	0.0404 *	5.0 ± 9.4
HbA1c (%)	6.1 ± 0.7	6.2 ± 0.8	0.74	0.073 ± 0.517
Apo B/Apo A1	0.65 ± 0.16	0.64 ± 0.16	0.11	−0.012 ± 0.081
HbA1c/Apo A1	0.053 ± 0.011 *	0.049 ± 0.011 *	0.0489 *	0.073 ± 0.517
Fasting blood glucose (mg/dL)	110.8 ± 20.4	116.6 ± 24.1	0.24	5.9 ± 22.5
2 h -OGTT blood glucose (mg/dL)	193.5 ± 78.9	182.5 ± 79.6	0.29	−11.0 ± 48.0
Rutherford classification	1.5 ± 0.6 ***	0.2 ± 0.4 ***	*p* *** < 0.001	−1.4 ± 0.8

*p* * < 0.05, *p* ** < 0.01, *p* *** < 0.001.

**Table 4:** Change in muscle area, apolipoprotein A1, apolipoprotein B, glucose metabolism, and Rutherford classification in gain or loss of skeletal muscle after EVT.

	Gain of Skeletal Muscle n = 12			Loss of Skeletal Muscle n = 10		
	Before	6 Months	*p*-Value	Before	6 Months	*p*-Value
Delta muscle area (cm^2^)	NA	8.41 ± 5.93	NA	NA	−4.67 ± 2.41	NA
Apo A1 (mg/dL)	121.8 ± 15.1 ***	136.5 ± 19.5 ***	*p* *** < 0.001	115.6 ± 20.1	121.4 ± 17.1	0.32
Apo B (mg/dL)	76.4 ± 19.2	80.5 ± 17.0	0.10	78.6 ± 19.8	82.3 ± 19.3	0.24
HbA1c (%)	5.8 ± 0.4	6.0 ± 0.4	0.97	6.5 ± 0.8	6.5 ± 1.1	0.50
Apo B/Apo A1	0.64 ± 0.17	0.60 ± 0.17	0.069	0.69 ± 0.19	0.68 ± 0.16	0.66
HbA1c/Apo A1	0.049 ± 0.007 **	0.045 ± 0.008 **	0.007 **	0.058 ± 0.004	0.054 ± 0.004	0.37
Fasting blood glucose (mg/dL)	110.2 ± 16.8	111.4 ± 22.2	0.83	111.5 ± 16.8	122.9 ± 25.9	0.094
2 h-OGTT blood glucose (mg/dL)	189.7 ± 67.5	170.6 ± 69.7	0.075	198.1 ± 94.4	196.7 ± 91.8	0.93
Rutherford classification	1.6 ± 0.5 ***	0.3 ± 0.5 ***	*p* *** < 0.001	1.5 ± 0.7 ***	0.1 ± 0.3 ***	*p* *** < 0.001

*p* ** < 0.01, *p* *** < 0.001, Not Applicable (NA).

## Data Availability

The raw data supporting the conclusions of this article will be made available by the authors on request. The data are not publicly available due to privacy.

## Supplementary Materials

The following supporting information can be downloaded at: https://www.mdpi.com/article/10.3390/metabo14040192/s1, Figure S1: The correlational matrix between delta values of Apo A1, B, fasting glucose, post 2 h-blood glucose, HbA1c, Rutherford classification, and delta thigh muscle area after EVT in skeletal muscle gain patients; Figure S2: The correlational matrix between delta values of Apo A1, B, fasting glucose, post 2 h-blood glucose, HbA1c, Rutherford classification, and delta thigh muscle area after EVT in skeletal muscle loss patients; Table S1: Statin regimen of the study cohort; Table S2: TREND Statement Checklist. Reference [46] is cited in the supplementary materials.
