# Supplementary material for: Exploring Possible Links: Thigh Muscle Mass, Apolipoproteins, and Glucose Metabolism in Peripheral Artery Disease—Insights from a Pilot Sub-Study following Endovascular Treatment
Source: Metabolites. 2024 Mar 29;14(4):192. doi: 10.3390/metabo14040192 (PMC11052193; doi:10.3390/metabo14040192)
Supplement: Supplementary file 1 [file metabolites-14-00192-s001.zip › Table S1.pdf]

# Supplemental Material

**Table S1. Statin regimen of the study cohort**

| | All | Gain of skeletal muscle | Loss of skeletal muscle | P-value |
|---|---|---|---|---|
| Number of patients | 22 | 12 | 10 | |
| Statin | 21 (95) | 11 (92) | 10 (100) | N. S |
| Rosuvastatin 5 mg | 3 (14) | 2 (17) | 1 (10) | N. S |
| Rosuvastatin 2.5 mg | 4 (18) | 2 (17) | 2 (20) | N. S |
| Atorvastatin 20 mg | 1 (4) | 1 (8) | 0 (0) | N. S |
| Atorvastatin 10 mg | 4 (18) | 2 (17) | 2 (20) | N. S |
| Atorvastatin 5 mg | 2 (9) | 0 (0) | 2 (20) | N. S |
| Pitavastatin 2 mg | 1 (4) | 1 (8) | 0 (0) | N. S |
| Pitavastatin 1 mg | 4 (18) | 2 (17) | 2 (20) | N. S |
| Pravastatin 10 mg | 2 (9) | 1 (8) | 1 (10) | N. S |

Data are presented as numbers (%). Not significant (N.S)
