# Supplementary figures and images for: Exploring Possible Links: Thigh Muscle Mass, Apolipoproteins, and Glucose Metabolism in Peripheral Artery Disease—Insights from a Pilot Sub-Study following Endovascular Treatment
Source: Metabolites. 2024 Mar 29;14(4):192. doi: 10.3390/metabo14040192 (PMC11052193; doi:10.3390/metabo14040192)

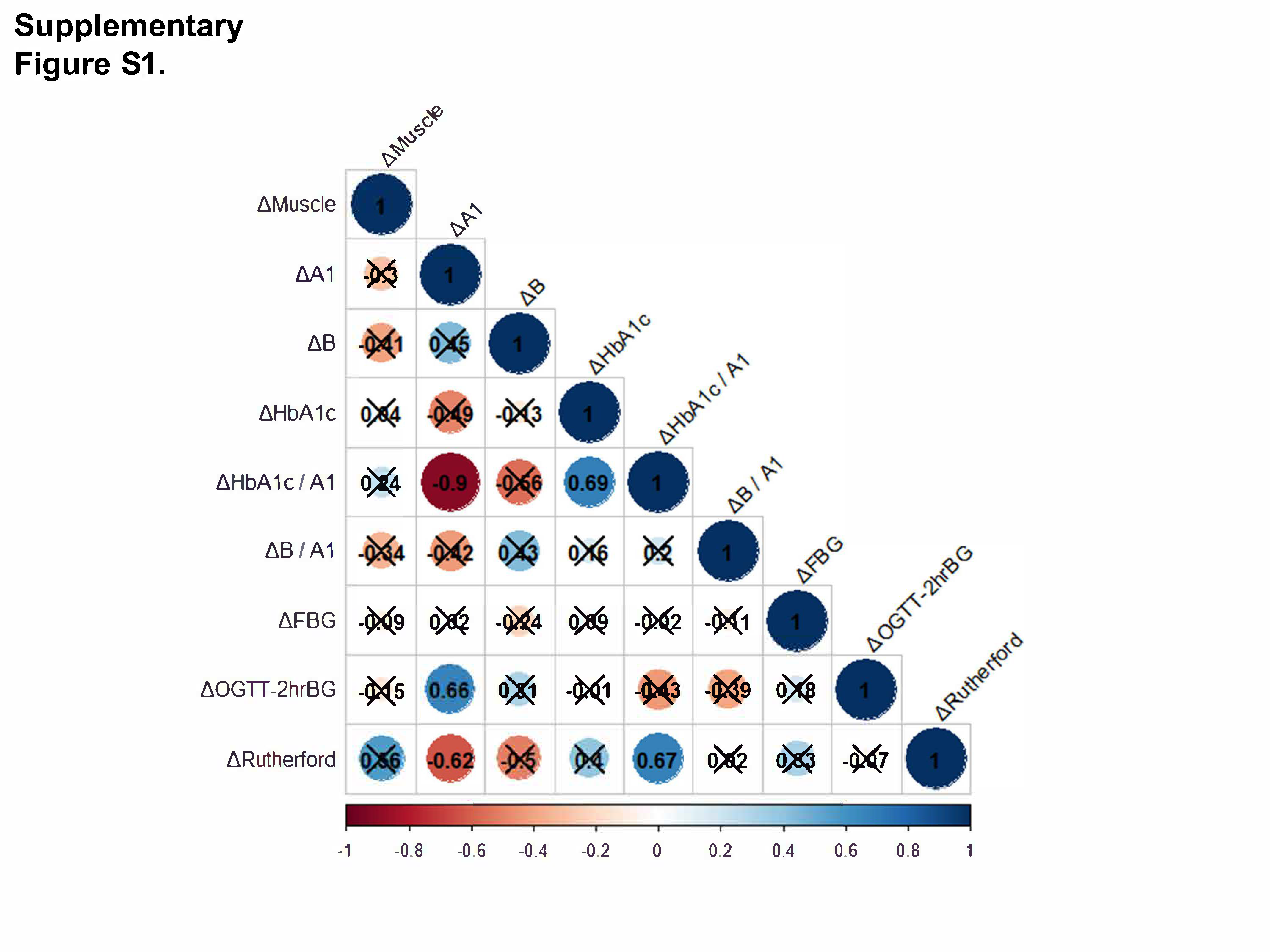

Supplement: Supplementary file 1 [file metabolites-14-00192-s001.zip › Figure S1.TIF]

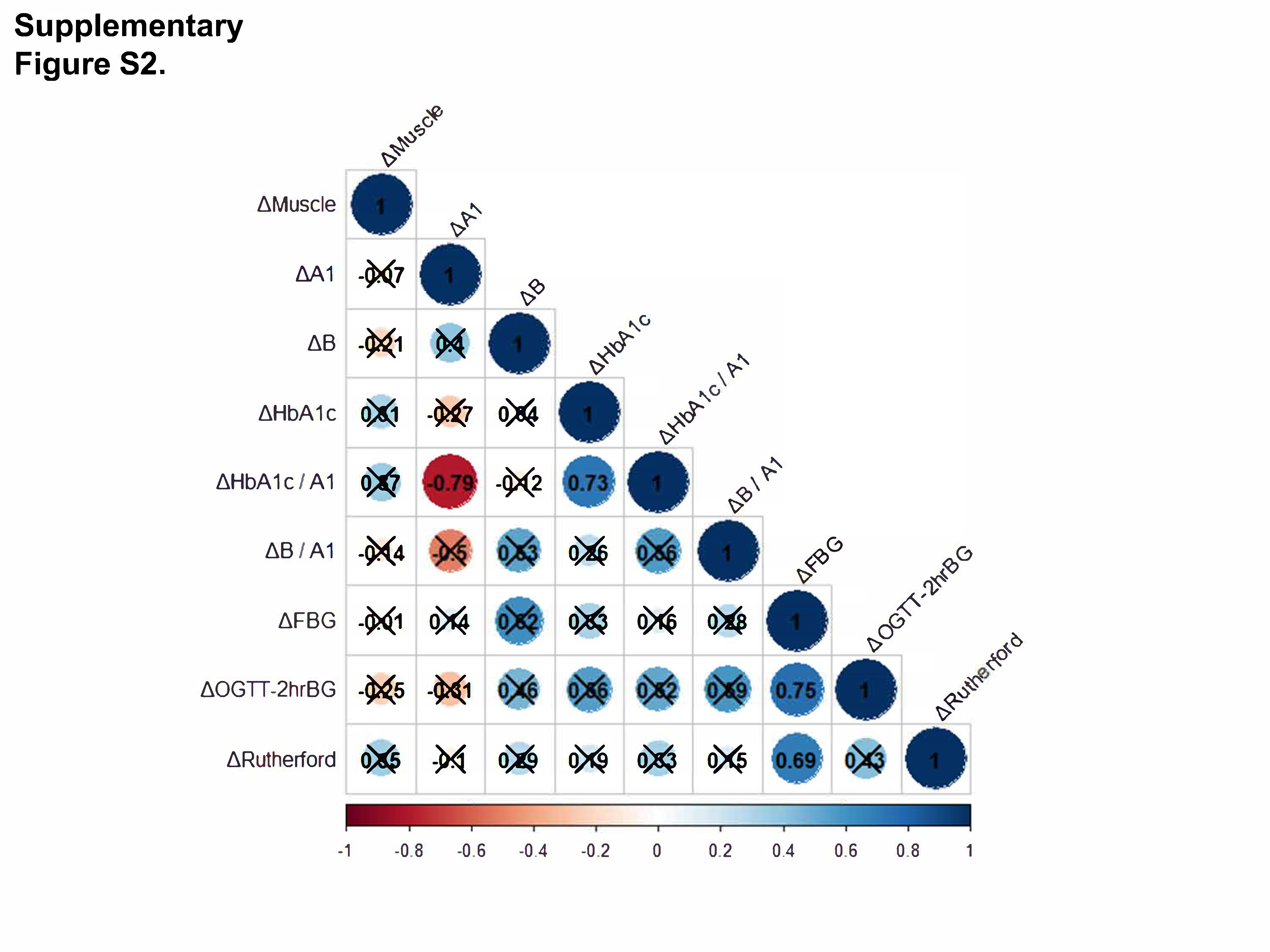

Supplement: Supplementary file 1 [file metabolites-14-00192-s001.zip › Figure S2.TIF]
